# Effects of Compound Active Peptides on Protecting Liver and Intestinal Epithelial Cells from Damages and Preventing Hyperglycemia

**DOI:** 10.1155/2020/3183104

**Published:** 2020-04-03

**Authors:** Xiaoxiang Xu, Yingjia Wei, Mian Khaqan Shah, Xiaoyu Wang, Junting Lin, Peng Wan, Li Cui, Qingqiang Yin

**Affiliations:** ^1^Shanghai Key Laboratory of Veterinary Biotechnology, College of Agriculture and Biology, Shanghai Jiao Tong University, Shanghai 200240, China; ^2^College of Animal Science and Veterinary Medicine, Henan Agricultural University, Zhengzhou 450002, China; ^3^Shanghai Fengni Medical Co., Ltd., Shanghai 200333, China

## Abstract

Active peptides have good effectiveness in controlling or preventing many diseases. Compound active peptides (CAP) obtained from animal, plant, and sea food proteins were used in this study to explore their effects on antioxidation, anti-inflammation, and antihyperglycemia *in vitro* and *in vivo*. The results demonstrated that 10 *μ*g/mL CAP could increase cell viability (*P* < 0.05) and decrease reactive oxygen species (ROS) levels and cell apoptosis (*P* < 0.05) when WRL68 cells were induced by H_2_O_2_ for 6 h. Moreover, incubation with 20 *μ*g/mL CAP for 6 h significantly increased cell viability and Bcl-2 expression level (*P* < 0.05) and decreased expression levels of IL-6, IL-8, TNF-*α*, Bax, and Caspase 3 and the ratio of Bax/Bcl-2 (*P* < 0.05) when swine jejunal epithelial cells (IPEC-J2) were induced by deoxynivalenol (DON). In addition, adding CAP individually or combined with Liuweidihuang pills (LDP, Chinese medicine) and low-dose glibenclamide could lower blood glucose levels in alloxan-induced hyperglycemic model mice. These results suggested that CAP was probably a beneficial ingredient for alleviating H_2_O_2_-induced oxidative stress and DON-induced cell inflammation and apoptosis and preventing hyperglycemia.

## 1. Introduction

Active peptides defined as specific regions of proteins with a part of 20 natural amino acid sequences in a certain order and structure have certain biological or physiological effects. Diverse types and orders of amino acids determine their extensive functions. Active peptides are extremely abundant in many resources, which can be extracted from animal or plant proteins such as wheat, rice, soybean, egg, and sea food [1–3]. Active peptides are generally named after their ingredient types, for example, soybean peptide, rice bran peptide, peanut peptide, sea cucumber peptide, and oyster peptide. Previous studies have demonstrated that active peptides possess multiple biological activities, including growth promotion, immune regulation, antioxidant, antihypertensive, antithrombotic, antiadipogenic, antimicrobial, and anti-inflammatory and immunomodulatory effects [4–8]. The different active peptides may have the different functions. In order to increase the effectiveness of CAP, many kinds of active peptides extracted from oysters, sea cucumbers, soybean, and other organisms were selected and mixed together in this study.

Oxidative stress represents an imbalance between oxidant and antioxidant agents, which will lead to inflammatory infiltration of neutrophils and produce a large number of oxidative intermediates. The reactive oxygen species (ROS) is a natural part of aerobic life, which is responsible for the manifestation of cellular functions ranging from signal transduction pathways, defensing against invading microorganisms to the promotion of growth or death [9]. ROS such as hydrogen peroxide and hydroxyl radicals with strong oxidizing properties is constantly generated from radiation, metabolism, or environmental pollution and then removed by the organisms rapidly to maintain dynamic balance. When this homeostasis is disturbed, excessive ROS can cause damage in the cellular and genetic levels, leading to cell apoptosis [10]. Previous studies indicated that extracellular H_2_O_2_ increased intracellular ROS levels via multiple mechanisms including the loss of intracellular ROS antioxidants such as glutathione (GSH) or the decrease of mitochondrial membrane permeability followed by mitochondrial ROS release [11, 12]. Therefore, H_2_O_2_ was employed to induce oxidative damage in liver cells in the current study. In addition to the well-known antioxidants such as vitamin C and vitamin E, some antioxidant peptides also have this character, which can reduce cell damage and promote cell repair. The previous research has shown that marine organisms have many antioxidants consisting of superoxide dismutase, peroxidase, and catalase as well as a variety of sterols [7]. CAP extracted from marine organisms such as sea cucumbers, scallops, and oysters also has antioxidative and antiaging attributes.

Deoxynivalenol (DON), also called as vomitoxin, is a common mycotoxin mainly produced by *Fusarium graminearum* and *Fusarium culmorum*, which often causes cytotoxicity and inflammation in humans and animals [13]. It was reported that DON could impair cell proliferation and viability and induce inflammation and apoptosis in swine jejunal epithelial cells (IPEC-J2) [14, 15]. In the present study, IPEC-J2 cells were used to establish DON-induced inflammation model to explore the protective effects of CAP.

Diabetes is a chronic metabolic disease characterized by hyperglycemia. It is often accompanied with a variety of serious complications such as blindness, cardiovascular disease, heart disease, nephropathy, amputation, and nerve damage. Although several synthesized drugs can effectively control the progression of diabetes and complications, they have some side effects. Therefore, finding safe natural products to replace drugs for alleviating diabetes has become more and more important. It was reported that some active peptides are effective to alleviate diabetes [16–18]. Generally, the continuous generation of free radicals in diabetic patients directly leads to the complications of hyperglycemia in various organs due to cellular damage. Therefore, if the oxidative system of the damaged cells is restored, it will contribute to attenuate the course of diabetes. The previous researches indicated that CAP had an antioxidant activity [6, 17, 19], so it is inferred that CAP may have benefits to alleviate diabetes. In order to explore the effective method for curing diabetes, the combined therapeutic effect of CAP and hypoglycemic drugs was also used in this study.

Because compound active peptides (CAP) have various biological functions, this research focused on its applications in antioxidation, anti-inflammation, and hyperglycemic treatment. At first, H_2_O_2_-induced WRL68 cells and DON-induced IPEC-J2 cells were used *in vitro* to make the cell-damage models to study CAP functions for alleviating oxidative stress, inflammation, and apoptosis. Secondly, alloxan-induced hyperglycemic model mice were used to preliminarily explore the relationship between diabetes and antioxidant activity of CAP. Through the experiments *in vivo* and *in vitro*, it will lay a theoretical foundation for CAP development and application.

## 2. Materials and Methods

### 2.1. Chemicals and Reagents

CAP was provided by Shanghai Fengni Pharmaceutical Technology Co., Ltd. (Shanghai, China). DON was purchased from Sigma-Aldrich (St. Louis, MO, USA), dissolved in dimethylsulfoxide (DMSO) (Sigma-Aldrich, St. Louis, MO, USA), formulated into 1 mg/mL stock solution, and stored at -20°C. H_2_O_2_, phosphate-buffered saline (PBS, pH = 7.4), 0.25% pancreatin with or without ethylenediaminetetraacetic acid (EDTA), penicillin-streptomycin, thiazolyl blue tetrazolium bromide (MTT), alloxan, and normal saline were purchased from Sigma-Aldrich (St. Louis, MO, USA). Dulbecco's Modified Eagle Medium (DMEM), high-glucose DMEM, and fetal bovine serum (FBS) were purchased from Thermo Fisher Scientific (Waltham, MA, USA). The reactive oxygen species (ROS) and Annexin V-FITC/PI cell apoptosis kit were purchased from Beyotime Bioengineering Institute (Shanghai, China). Glibenclamide was purchased from Meryer Chemical Technology Co., Ltd. (Shanghai, China). Liuweidihuang pill was purchased from Beijing Tongrentang Co., Ltd. (Beijing, China).

### 2.2. Compound Active Peptide Composition and Detection

CAP used in this study included soybean peptide, ginseng peptide, bovine colostrum peptide, sea cucumber peptide, and oyster peptide. The separation and purification of CAP were carried out by column chromatography with parameters of 1.9 *μ*m film thickness, 0.075 mm inner diameter, and 250 mm column length (ReproSil-Pur C18-AQ, Maccura, Germany). It was measured by liquid chromatograph (EASY-nLC 1200, Thermo, USA). Ground solid sample (about 0.7 g) was completely dissolved in 1 mL 8 mol/L guanidine hydrochloride and 50 mM PBS and then subjected to 10-fold dilution and desalting treatment. Column chromatography and mass spectrometry were performed as follows: 5 *μ*L sample was injected with auto-sampler into the column at 45°C; the stationary phase was silica gel; flow phase A was deionized water (containing 0.1% fatty acid); flow phase B was 80% acetonitrile (containing 0.1% fatty acid); and the flow rate was 0.3 *μ*L/min. The flow phase gradient settings are listed in Table 1. After mass spectrometry analysis, the analytical alignment was performed using PEAKS studio software to obtain the sequence analysis and posttranslational modification of different peptides in the sample. Sequence analysis of the peptide chain was performed by a mass spectrometer (Q Exactive Plus Orbitrap MS, Thermo, USA).

### 2.3. Cell Culture and Treatments

Human hepatic cell line (WRL68) was obtained from Shanghai Key Laboratory of Veterinary Biotechnology, China. IPEC-J2 cell line was obtained from the College of Animal Science and Technology, Jiangxi Agricultural University, China. The WRL68 cells and IPEC-J2 cells were cultured at 37°C in an incubator with 5% CO_2_ in DMEM and high-glucose DMEM supplemented with 10% FBS and 1% penicillin-streptomycin, respectively. The cells were cultured for 24 h before subjecting to different treatments. The treatment solutions were diluted with complete medium without serum and antibiotics. WRL68 cells were grouped into control, H_2_O_2_ (10, 50, 100, 200, and 500 *μ*mol/L H_2_O_2_, incubated for 6 h), H_2_O_2_ 6 h+0.1 *μ*g/mL CAP 24 h, H_2_O_2_ 6 h+1 *μ*g/mL CAP 24 h, and H_2_O_2_ 6 h+10 *μ*g/mL CAP 24 h. IPEC-J2 cells were treated with the control group, DON group (0.5 *μ*g/mL), CAP group (10, 20, 40, 80 *μ*g/mL), and CAP+DON group for 6 h.

### 2.4. Cell Viability Assay

Cell viability was performed using MTT assay. WRL68 cells were seeded into 96-well plates at a density of 6 × 10^3^ cells/well, and IPEC-J2 cells were seeded into 96-well plates at a density of 1 × 10^4^ cells/well. All cells were cultured for 24 h, followed by removing the culture medium and washing twice with PBS, and subsequently incubated with different treatments. At the end of the treatments, each well was added with 10 *μ*L of 5 mg/mL MTT and incubated for 4 h at 37°C. Then, the cell supernatant was removed and 150 *μ*L DMSO was added to each well. The plate was shaken at room temperature for 10 min. The optical density (OD) was measured at a wavelength of 490 nm using an enzyme-linked immunosorbent assay. The cell viability (%) was calculated as OD_490_ value in experimental group/OD_490_ value in control group × 100%.

### 2.5. Intracellular ROS Concentration and Apoptosis Analysis

WRL68 cells were seeded into 12-well plates at a density of 1 × 10^5^ cells/well. After culturing for 24 h, the cells were grouped into control, H_2_O_2_, and H_2_O_2_+CAP groups. The H_2_O_2_ group was treated with 200 *μ*mol/L H_2_O_2_ for 6 h and H_2_O_2_+CAP groups were pretreated with 200 *μ*mol/L H_2_O_2_ for 6 h and then treated with different concentrations of CAP for another 24 h. After the cells were digested with 0.25% trypsin, they were centrifuged, and then, 300 *μ*L of serum-free medium containing DCFH-DA (10 *μ*M) was added. The cells were resuspended and incubated at 37°C for 20 min and then washed three times with serum-free medium. ROS levels were measured by flow cytometry at an excitation wavelength of 488 nm and an emission wavelength of 525 nm. Apoptotic cells were detected using an Annexin V-FITC/PI kit according to the manufacturer's instructions.

The cells were harvested into 5 mL EP tubes and washed twice with the incubation buffer. Subsequently, the cells were centrifuged and resuspended with 100 *μ*L 1x binding buffer and then stained with 5 *μ*L Annexin V-FITC and 5 *μ*L PI at room temperature in the dark for 10-15 min. The cells were centrifuged and washed with incubation buffer. Furthermore, the fluorescent (SA-FLOUS) solution was added, and the cells were incubated at 4°C for 20 min in the dark. Finally, cell apoptosis was analyzed by flow cytometry at the excitation wavelength of 488 nm, and the FITC fluorescence was detected by a passband filter with a wavelength of 515 nm. PI was detected by another filter with a wavelength of more than 560 nm. The results were determined by a bivariate flow cytometry scatter plot. Q1, Q2, Q3, and Q4 represented necrotic cell rates, late apoptotic cell rates, early apoptotic cell rates, and viable cell rates, respectively.

### 2.6. RNA Extraction and Quantitative Real-Time PCR

IPEC-J2 cells were seeded with a density of 5 × 10^5^ cells/well in 6-well culture plates and cultured for 24 h. Then, the cells were treated with control, 0.5 *μ*g/mL DON, 20 *μ*g/mL CAP, and 20 *μ*g/mL CAP+0.5 *μ*g/mL DON for 6 h. After reaction, total RNA was extracted using a Trizol reagent. RNA was dissolved in 40 *μ*L RNase-free water and stored at -80°C. cDNA was reverse-transcribed from RNA using a TB GREEN kit (TaKaRa, Dalin, China). Quantitative real-time PCR was performed using CFX Connect™ Real-Time PCR Detection System (Bio-Rad, Hercules, CA, USA). The primers synthesized by Shenggong Biotech Co., Ltd. (Shanghai, China) are listed in Table 2. The relative mRNA abundances were calculated by the 2^-∆∆Ct^ method in comparison with the expression levels of GAPDH.

### 2.7. Animals

All the experiments including animal housing and sacrificing were approved by the Animal Ethical Committee of Shanghai Jiao Tong University (A2019089) prior to the beginning of the experiment. Male KM mice with 18-22 g body weight (BW) were obtained from the Laboratory Animal Center in Shanghai Jiao Tong University, China. All the mice were housed with 12 h light and 12 h dark. Food and water were given *ad libitum*. The mice were subsequently divided into two groups. The control mice (*n* = 6) were fed a normal diet, and the model mice (*n* = 39) were intraperitoneally injected with 90 mg/kg BW alloxan after fasting for 12 h. Blood glucose was measured by a fast blood glucose tester after 72 h (fasting for 4 h before measurement). The blood glucose concentration between 11 and 30 mmol/L was considered to be a successful model, which could be used in the subsequent experiments. The experiments were divided into two parts. One part was the single medication experiment (*n* = 15) for 14 d, including 5 groups (three mice in each group) such as the control group (CG), the model group (MG), 20 mg/kg BW/d glibenclamide treatment group (GLI), 0.4 g/kg BW/d Liuweidihuang pill treatment group (LDP), and 1.2 g/kg BW/d compound active peptide treatment group (CAP). Another part was the combined medication experiment (*n* = 30) including 10 experimental groups (three mice in each group): CG: control group (normal saline), MG: model group (normal saline), GT: 10 mg/kg BW/d glibenclamide treatment, GAP: glibenclamide+CAP, GLDP: glibenclamide+Liuweidihuang pill treatment, TCD: treatment combined with 3 kinds of drugs, DMG: degressive glibenclamide, APG: CAP+degressive glibenclamide, LDG: Liuweidihuang pill+degressive glibenclamide, and ALG: CAP+Liuweidihuang pill+degressive glibenclamide. The mice in the control and the model groups were intragastrically administered with the same volume of normal saline as the other groups once a day for 31 d, and the intragastric volume was 0.01 mL/g BW/d. The specific periods of combined dosing and descending dosing are listed in Table 3.

### 2.8. Detection Indexes

All the mice were fasted for 1 d before blood sampling. In the single medication experiment, blood glucose concentrations were detected in the morning on the 0, 7th, and 14th d. In the combined medication experiment, blood glucose concentrations were detected on the 0, 14th, 21st, 28th, and 31st d. In order to investigate the healing of the wounded tails after different treatments, healing situation of mice tails was observed and recorded on the 31st day before blood collection.

### 2.9. Statistics and Analysis

All the data were expressed as mean ± standard deviation. Statistical analysis was analyzed by one-way ANOVA with a Duncan multiple comparison test using SPSS 20.0 software (Sishu Software, Shanghai Co., Ltd., Shanghai, China). All graphs were generated using GraphPad Prism 8 (GraphPad Software, La Jolla, USA). The differences were regarded as a statistically significant difference at *P* < 0.05.

## 3. Results

### 3.1. CAP Alleviating H_2_O_2_-Induced WRL68 Cell Damage

**Figure 1:**
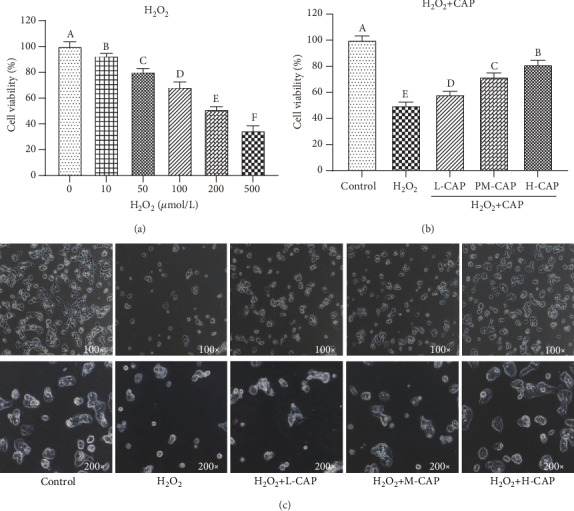
CAP alleviating H_2_O_2_-induced WRL68 cell damage (*n* = 6). Note: (a) the viability of WRL68 cells induced by different H_2_O_2_ concentrations. (b) The cell viability after H_2_O_2_ inducement and treatment with different concentrations of CAP. (c) The cell morphology after H_2_O_2_ inducement and treatment with different concentrations of CAP (magnification 100x and 200x). Different letters on the bars represent a significant difference from each other (*P* < 0.05), while the same letters on the bars represent an insignificant difference from each other (*P* > 0.05). L-CAP: low concentration of compound active peptides (0.1 *μ*g/mL); M-CAP: middle concentration of compound active peptides (1 *μ*g/mL); H-CAP: high concentration of compound active peptides (10 *μ*g/mL). The same as below.

The cells were seriously damaged, and the cell survival rates were significantly decreased with increasing H_2_O_2_ concentration (*P* < 0.05, Figure 1(a)). About 50% cell viability was usually selected as the optimal modeling condition for cell damage standard. Therefore, the model group treated with 200 *μ*mol/L H_2_O_2_ was used in the subsequent experiments. Compared with the H_2_O_2_ injury group (49.15%), adding high concentrations of CAP to H_2_O_2_ injury cells could significantly increase cell viability by 83.96% (*P* < 0.05). At the same time, the morphology of cells was observed by inverted phase contrast microscopy (Figure 1(b)). The number of intact cells in the H_2_O_2_ group was significantly lower than that in the control group; however, CAP addition was able to increase the number of intact cells induced by H_2_O_2_ (Figure 1(c)), corresponding with the results of cell viability. The higher the CAP concentration, the higher the number of intact cells.

### 3.2. CAP Inhibited Intracellular ROS Accumulation and Apoptosis in WRL68 Cells

**Figure 2:**
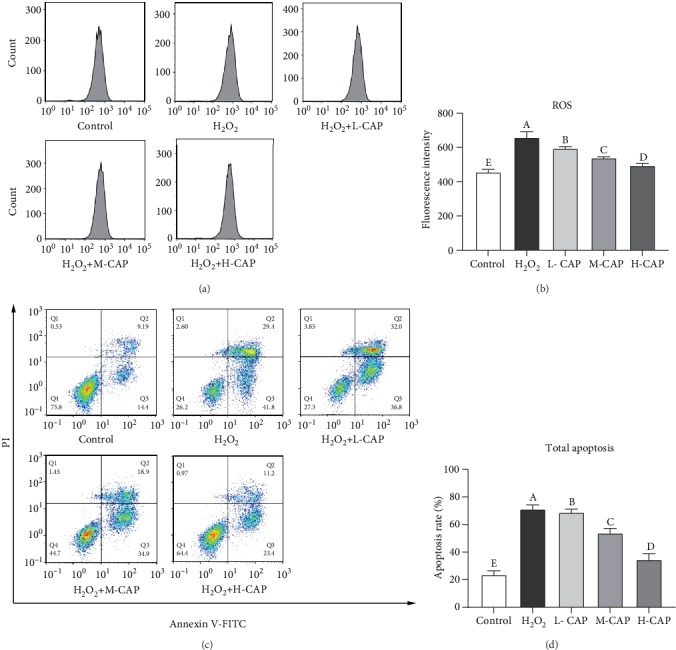
The protection of CAP against H_2_O_2_-induced ROS accumulation and apoptosis in the WRL68 liver cells (*n* = 3). Note: (a) the flow cytometric analysis of ROS count. (b) The fluorescence intensity of the cells calculated relative to the control group. (c) Flow cytometry analysis of Annexin V/FITC/PI staining cells; Q1 indicates necrotic cell rates, Q2 indicates later apoptotic cell rates, Q3 indicates early apoptotic cell rates, and Q4 indicates viable cell rates. (d) The flow cytometric analysis of apoptotic cell rates. Different letters on the bars represent a significant difference from each other (*P* < 0.05), while the same letters on the bars represent an insignificant difference from each other (*P* > 0.05).

The intracellular ROS was significantly increased in the H_2_O_2_ injury group (*P* < 0.05). However, treatment with different doses of CAP for 24 h decreased the accumulation of intracellular ROS induced by H_2_O_2_, and the ROS levels were decreased with the increasing CAP dose (Figures 2(a) and 2(b), *P* < 0.05).  Figure 2(c) showed that the necrotic, later apoptotic, early apoptotic, and viable cell rates were 0.59%, 9.16%, 15.37%, and 74.83% for the control group; 2.72%, 28.9%, 42.9%, and 25.5% for the H_2_O_2_-induced group; 4.07%, 31.5%, 37.73%, and 26.67% for the H_2_O_2_+L-CAP group; 1.59%, 18.53%, 36.2%, and 43.67% for the H_2_O_2_+M-CAP group; 1.07%, 10.93%, 24.37%, and 63.63% for the H_2_O_2_+H-CAP group. As shown in Figure 2(d), H_2_O_2_ injury significantly increased the total apoptotic cell rates (*P* < 0.05); however, CAP addition could significantly decrease the apoptotic cell rates (*P* < 0.05). This result indicated that high concentration of CAP could significantly decrease cell apoptosis and increase cell viability under the condition of H_2_O_2_ inducement.

### 3.3. CAP Alleviated DON-Induced Cytotoxicity in IPEC-J2 Cells

**Figure 3:**
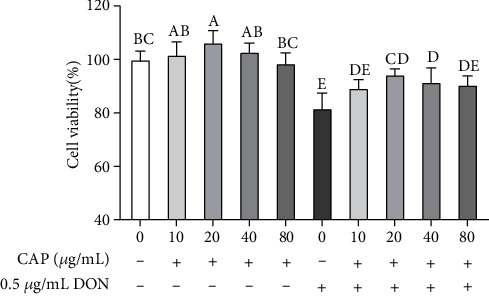
CAP alleviating DON-induced cytotoxicity in IPEC-J2 cells (*n* = 3). Note: different letters on the bars represent a significant difference from each other (*P* < 0.05), while the same letters on the bars represent an insignificant difference from each other (*P* > 0.05).

As shown in Figures 3, 20 *μ*g/mL CAP significantly increased cell viability (*P* < 0.05), compared with the control group. 0.5 *μ*g/mL DON significantly decreased cell viability (*P* < 0.05); however, 20-40 *μ*g/mL CAP alleviated cell damage induced by DON (*P* < 0.05). Therefore, 20 *μ*g/mL CAP was used in the subsequent cell experiments.

### 3.4. CAP Alleviating IPEC-J2 Cell Inflammation and Apoptosis Induced by DON

**Figure 4:**
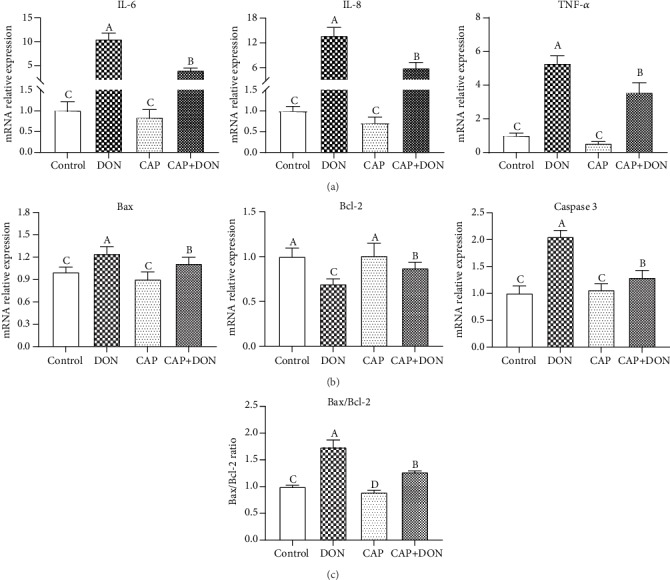
Effect of CAP on alleviating inflammation and apoptosis in DON-induced IPEC-J2 cells (*n* = 3). Note: (a) the mRNA expressions of inflammation-related genes; (b) the mRNA expressions of apoptosis-related genes; (c) the ratio of Bax/Bcl-2. Different letters on the bars represent a significant difference from each other (*P* < 0.05), while the same letters on the bars represent an insignificant difference from each other (*P* > 0.05).

The relative mRNA expressions of IL-6, IL-8, TNF-*α*, Bax, and Caspase 3 and the Bax/Bcl-2 ratio in IPEC-J2 cells were significantly upregulated (*P* < 0.05), and the B-cell lymphoma-2 (Bcl-2) mRNA abundance was significantly downregulated by DON exposure for 6 h (*P* < 0.05, Figure 4). However, compared with the single DON treatment, IL-6, IL-8, TNF-*α*, Bax, and Caspase 3 mRNA expressions and the Bax/Bcl-2 ratio were significantly decreased, while Bcl-2 mRNA abundance was significantly increased by CAP addition (*P* < 0.05). These results preliminarily inferred that CAP could alleviate the cell inflammation and apoptosis induced by DON.

### 3.5. CAP Combined with Glibenclamide Reduced Blood Glucose Content in Alloxan-Induced Hyperglycemia Model Mice

**Figure 5:**
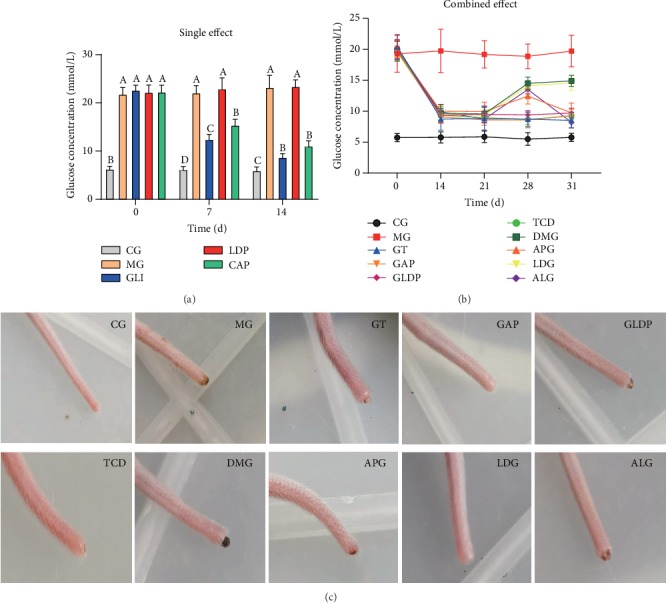
Hypoglycemic action in single and combined medication experiment in alloxan-induced hyperglycemia model mice (*n* = 3). Note: (a) effect of a single factor on the levels of blood glucose; (b) effect of combined factors on the levels of blood glucose; (c) the repairment of the wounded tails after treatment with different drugs. Different letters on the bars represent a significant difference from each other (*P* < 0.05), while the same letters on the bars represent an insignificant difference from each other (*P* > 0.05).

It was shown that administering CAP or glibenclamide (GLI) for 14 d significantly reduced the levels of blood glucose in hyperglycemia mice (*P* < 0.05), compared with the model group (MG), indicating that CAP had the same function as glibenclamide to decrease blood glucose concentration. The blood glucose content in the group treated with LDP was close to that in the model group, which indicated that the utilization of LDP alone had no effect to decrease blood glucose concentration (Figure 5(a)). The combined hypoglycemic effects of GAP, TCD, and ALG and other combined factors are investigated in Figure 5(b). In the first 14 d, glibenclamide treatment (GT) had a significant effect on lowering blood glucose (below 11 mmol/L) (*P* < 0.05), but the blood glucose levels had no significant changes after adding other drugs. 21 d later, the GT doses of the four groups began to decrease by 1 mg/kg BW/d until day 28, and the blood glucose began to increase with the dose of gradually reducing GT. In particular, the blood glucose content was increased in LDG and DMG groups. Moreover, in the CAP-containing groups such as GAP, TCD, and ALG, the blood glucose levels were slightly lower, which was almost the same as the simple TG group. There was a significant difference between the GT degressive group and the model group (*P* < 0.05). These results demonstrated that CAP had a hypoglycemic effect to reduce glibenclamide dosage by combining with other medicines under the context of maintaining therapeutic effects. Furthermore, at day 31, mouse tails in GAP, TCD, and LDG groups were almost healed with a healthy pink color. The pictures also indicated that CAP can accelerate the repair of the wounded tails more effectively (Figure 5(c)).

## 4. Discussion

Oxidative stress is one of the important causes of apoptosis [19, 20]. Oxidative stress caused by ROS is responsible for a wide variety of cellular damages and is also the most validated mechanism of secondary injury [21]. ROS including hydrogen peroxide (H_2_O_2_), superoxide anion (O^2−^), and hydroxyl radical (OH^−^) are physiologically produced at a basal rate. H_2_O_2_ has been widely used as model exogenous oxidative stress-mediated experiment in liver cells [22, 23]. In this research, H_2_O_2_ significantly decreased the viability of WRL68 cells as well as increased the ROS levels and apoptotic cell rates. Currently, some synthesized antioxidants are used to prevent oxidative damage, but they are progressively discarded due to their potential risks to human health. Active peptides have been reported to have antioxidant activity similar to or better than synthetic antioxidants [24, 25]. Liu et al. showed that polypeptide from *Chlamys farreri* (PCF) had strong antioxidant activity, which could reduce the intracellular ROS production and protect UVB-induced HaCaT cell apoptosis [26]. This study indicated that CAP addition could alleviate the above symptoms of WRL68 cells induced by H_2_O_2_. Furthermore, there was a significant correlation between the ROS fluorescence intensity and cell viability. It can be speculated that CAP may have an antioxidant function to reduce the level of oxidative stress of cells, resulting in low cell apoptosis and high cell viability.

Deoxynivalenol (DON) is one of the most prevalent mycotoxins, which is frequently detected in various foods, feeds, and farm animals. The intestinal epithelium cells are the main targets of DON. The effect of DON on reducing the intestinal cell viability and inducing inflammatory and apoptosis has been proven in the previous study [15]. IL-6, IL-8, and TNF-*α* were the three most representative inflammatory cytokines, and their increasing expression levels indicated an inflammatory response. Caspase 3 is an important cytoplasmic enzyme; once it was activated, the occurrence of apoptosis was irreversible [27]. Bcl-2 belongs to the family of antiapoptosis, and Bax can promote apoptosis; therefore, the high value of Bax/Bcl-2 is usually used as an indicator of apoptosis [28]. CAP addition can decrease cytokine gene expressions and Bax/Bcl-2 value, inferring that CAP is able to alleviate inflammation and apoptosis in DON-induced IPEC-J2 cells.

Diabetes is a common metabolic disease characterized by hyperglycemia. The development of diabetes-curing drugs is a hot spot [29, 30]. It was reported that oxidative stress affected the progression of diabetes mainly by the following two mechanisms [10]: (1) directly cause islet *β*-cell damage through disrupting mitochondrial structure, causing apoptosis, inhibiting energy metabolism, reducing insulin synthesis and secretion, affecting the normal function of islet *β*-cells, etc and (2) induction of insulin resistance through interfering with phosphorylation of insulin receptors and their substrates, affecting cell signaling pathways [31]. Injection of alloxan in mice is usually used in modelling hyperglycemia, which is similar to the diabetic patients. It has been proved that metabolism of alloxan yields free radicals to induce cytotoxicity towards pancreatic-*β* cells [32]. The continuous generation of free radicals in diabetic patients directly leads to the complications of hyperglycemia in various organs due to cellular damage. Therefore, if the oxidative system of the damaged cells is restored, it will help to attenuate the course of diabetes. Active peptides have advantages of rapid transport and absorption, which contribute to the proliferation of cells and rapid reconstruction of tissue [33, 34]. It was also reported that the combined peptides isolated from a pumpkin had antioxidant and hypoglycemic activities [35]. This result indicated that CAP had certain antioxidant activity, so the hypoglycemic effect of CAP was explored through a diabetic mouse model *in vivo*. Glibenclamide is a second-generation sulfonylurea hypoglycemic agent, which is one kind of common drugs for the clinical treatment of diabetes. However, it has some side effects such as renal dysfunction and hypoglycemia. Therefore, if the dose of glibenclamide can be reduced by a combination of other drugs, its side effect will be decreased. Liuweidihuang pills (LDP), a traditional Chinese medicine, have been proved to have a good effect on diabetes. Pharmacological tests have shown that LDP has antioxidant, insulin resistance improving, antiapoptosis, and other effects [36]. Therefore, LDP was selected in this experiment to explore its hypoglycemic effect.

This result showed that CAP had almost the same hypoglycemic effect as glibenclamide when they were administered alone for 14 d, indicating CAP hypoglycemic function. In the combined drug experiment, CAP and LDP did not affect the efficacy of glibenclamide. When the dose of glibenclamide was decreased, its hypoglycemic effect in the DMG group became weak. However, the hypoglycemic effect in the ALG group recovered to almost the same level as the high-dose glibenclamide treatment (GT) group when low-dose glibenclamide treatment (DMG) was combined with CAP+LDP. This finding indicates that there is a cooperation among CAP, LDP, and low-dose glibenclamide to reduce the blood glucose level when the administered glibenclamide dose is low, which will help to reduce the side effect of glibenclamide. This is very important for developing more safe, efficient, and convenient treatments for diabetes.

## 5. Conclusions

Based on the results of cell models and animal experiments *in vitro* and *in vivo*, it was concluded that CAP could protect hepatic and intestinal cells from inflammation, apoptosis, and damages induced by the hazards such as toxins, oxidants, and stresses. CAP was also able to decrease blood glucose levels to treat hyperglycemia when it was used alone or combined with LDP and low-dose glibenclamide. These findings indicate that CAP is a promising bioactive substance in antioxidation, anti-inflammation, and antihyperglycemia; it may have potential in developments and applications of function food, feed additives, and alternative of hypoglycemic drugs.

## Figures and Tables

**Table 1:** Gradient setting of flow phase for column chromatography.

Time (min)	Flow rate (*μ*L/min)	Percentage of phase A	Percentage of phase B
0	0.3	95	5
2	0.3	92	8
42	0.3	77	23
50	0.3	60	40
52	0.3	0	100
60	0.3	0	100

**Table 2:** Primer sequences of genes for quantitative real-time PCR.

Gene	Accession number	Primer sequence (5′-3 ′)	Size (bp)
GAPDH	XM-004387206	F: ATGACCACAGTCCATGCCATC	271
		R: CCTGCTTCACCACCTTCTTG	

IL-6	NM_214399	F: GCTCTCTGTGAGGCTGCAGTTC	107
		R: AAGGTGTGGAATGCGTATTTATGC	

IL-8	NM_213867	F: GACCCCAAGGAAAAGTGGGT	136
		R: TGACCAGCACAGGAATGAGG	

TNF-*α*	NM_214022	F: TTCCAGCTGGCCCCTTGAGC	146
		R: GAGGGCATTGGCATACCCAC	

Bax	XM-003355975.1	F: ATGATCGCAGCCGTGGACACG	296
		R: AASTAGATGGTCACCGTCTGC	

Bcl-2	XM-003122573.2	F: AGAGCCGTTTCGTCCCTTTC	270
		R: GCACGTTTCCTAGASTGCAT	

Caspase 3	NM-214131.1	F: TTGGACTGTGGGATTGAGACG	165
		R: CGCTGCACAAAGTGACTGGA	

Note: GAPDH: glyceraldehyde-3-phosphate dehydrogenase; IL-6: interleukin 6; IL-8: interleukin 8; TNF-*α*: tumor necrosis factor *α*; Bcl-2: B-cell lymphoma-2; Bax: Bcl-2-associated X protein.

**Table 3:** Design of the combined dosing and descending dosing.

Groups	Treatment process
CG	Control group with normal saline for 31 d
MG	Model group with normal saline for 31 d
GT	Glibenclamide treatment (10 mg/kg BW/d) for 31 d
GAP	GT plus CAP (1.6 g/kg BW/d) added for the posterior 17 d
GLDP	GT plus LDP (0.4 g/kg BW/d) added for the posterior 17 d
TCD	GT plus (CAP+LDP) added for the posterior 17 d
DMG	GT for 21 d, decreased by 1 mg/kg BW/d for 7 d, and then kept at 3 mg/kg BW/d for 3 d
APG	DMG plus CAP added for the posterior 17 d
LDG	DMG plus LDP added for the posterior 17 d
ALG	DMG plus (CAP+LDP) added for the posterior 17 d

Note: except for the control group, diabetes model mice were used in the other groups. CAP: compound active peptide; LDP: Liuweidihuang pills (Chinese medicine).

## Data Availability

The data used to support the findings of this study are included within the article.
